# A High Throughput Protein Microarray Approach to Classify HIV Monoclonal Antibodies and Variant Antigens

**DOI:** 10.1371/journal.pone.0125581

**Published:** 2015-05-04

**Authors:** Emmanuel Y. Dotsey, Andrea Gorlani, Sampat Ingale, Chad J. Achenbach, Donald N. Forthal, Philip L. Felgner, Johannes S. Gach

**Affiliations:** 1 Division of Infectious Diseases, University of California Irvine, Irvine, California, United States of America; 2 Department of Cell and Molecular Biology, The Scripps Research Institute, La Jolla, California, United States of America; 3 Division of Infectious Diseases, Northwestern University, Chicago, Illinois, United States of America; Emory University, UNITED STATES

## Abstract

In recent years, high throughput discovery of human recombinant monoclonal antibodies (mAbs) has been applied to greatly advance our understanding of the specificity, and functional activity of antibodies against HIV. Thousands of antibodies have been generated and screened in functional neutralization assays, and antibodies associated with cross-strain neutralization and passive protection in primates, have been identified. To facilitate this type of discovery, a high throughput-screening tool is needed to accurately classify mAbs, and their antigen targets. In this study, we analyzed and evaluated a prototype microarray chip comprised of the HIV-1 recombinant proteins gp140, gp120, gp41, and several membrane proximal external region peptides. The protein microarray analysis of 11 HIV-1 envelope-specific mAbs revealed diverse binding affinities and specificities across clades. Half maximal effective concentrations, generated by our chip analysis, correlated significantly (P<0.0001) with concentrations from ELISA binding measurements. Polyclonal immune responses in plasma samples from HIV-1 infected subjects exhibited different binding patterns, and reactivity against printed proteins. Examining the totality of the specificity of the humoral response in this way reveals the exquisite diversity, and specificity of the humoral response to HIV.

## Introduction

The envelope glycoproteins gp41 and gp120, located on the surface of human immunodeficiency virus-1 (HIV-1), represent major targets for antibody recognition. Virus neutralization is predominantly mediated by antibodies binding to conserved regions on the native envelope trimer (Env), which is comprised of three non-covalently bound gp120/gp41 heterodimers. Characteristic envelope regions vulnerable to virus neutralization include the variable loops V1, V2, and V3, the CD4 binding site (CD4bs), certain N-linked oligomannose glycans on gp120, the membrane proximal external region (MPER) on gp41, and a recently discovered, but undefined target located in the gp41/gp120 interface of native Env [1–4]. Prominent neutralizing antibodies directed against these conserved regions are: 1) PG9, PG16, CH01-04, and PGT141-145 against the V1/V2 loop on gp120, 2) b12, VRC01, NIH45-46, 3BNC117, HJ16, 1F7, VRC-CH31, and VRC-PG04 against the CD4bs, 3) 2G12, PGT121-137 against a V3-specific glycan cluster on gp120, and 4) 10E8, 2F5, 4E10, and Z13e1 against the MPER on gp41 [5, 6]. All these neutralizing antibodies are characterized by high potency and cross clade recognition [6]. In contrast, non-neutralizing antibodies interact predominantly with non-functional Env (e.g. gp41 stumps, monomeric gp41/gp120 heterodimers, and uncleaved gp160 precursors)[7].

Anti—HIV antibodies are also able to mediate Fc effector functions such as, antibody dependent cellular cytotoxicity, or antibody dependent cellular phagocytosis [8, 9]. These may be important mechanisms for controlling viral load, and mediating protection [10–12]. Effector functions are triggered after viral antigens are opsonized by antibody, and their Fc domain cross-links Fc receptors, located on the surface of effector cells, such as natural killer (NK) cells, macrophages, and dendritic cells. Several groups showed that HIV-1 infected cells are effectively targeted and killed by NK cells after antibody opsonization [13–16]. It is thus important to evaluate antibody-binding characteristics of not only broadly neutralizing, but also non-neutralizing antibodies from different isolates and clades. Unfortunately, screening methods available today, e.g. ELISA, are generally elaborate, time consuming, and utilize large quantities of valuable specimens.

Recent advances in protein microarray technology have led to the development of proteome-wide pathogen-specific microarrays, allowing for robust, quantitative, and high throughput-screening of specific antibody responses in infected patients, while sparing valuable patient specimens [17–20]. Here we have successfully implemented this methodology to characterize HIV-1 specific antibody binding profiles against envelope glycoproteins, and derivatives, in a rapid and high throughput fashion. We evaluated a total of 15 HIV-1 specific mAbs against 15 HIV-1 multi-clade envelope proteins and gp41 MPER analogs, spotted at three different concentrations onto a microarray chip. We found substantial differences in the antibody-binding pattern of the tested antibodies. Further, we were able to calculate half maximal effective concentrations (EC_50_) and affinity constants by titrating antibody concentration on the chip by serial dilution. We observed a significant correlation between EC_50_ values generated by our microarray analysis when compared to EC_50_ values generated by standard binding ELISA, making the chip a more desirable tool for antibody characterization. Moreover, we screened human plasma samples of individuals with chronic HIV-1 infection on suppressive antiretroviral therapy (ART) to study the humoral immune response to HIV-1 *in vivo* and evaluate potential clinical applications. Beside a diverse antibody-binding pattern, we found a significant correlation between antibody titers (clade B) and neutralization, as well as cross clade reactivity and neutralization, thus highlighting the functionality of this chip analysis.

## Materials and Methods

### Ethic statement

This research was approved by the Institutional Review Boards at the Northwestern University. Subjects, from whom specimens were collected for study purposes, provided written informed consent. All experiments were performed in accordance with relevant guidelines and regulations.

### Antibodies, proteins, and peptides

MAbs 2G12, 4E10, 2F5, and 5F3 were kindly provided by Dietmar Katinger (Polymun Scientific). MAbs b12, Den3, and Z13e1 were gifts from Dennis R. Burton and Michael B. Zwick. John Mascola kindly provided VRC01 and VRC03. All other HIV-1 specific mAbs used in this study were obtained through the NIH AIDS Reagent Program, Division of AIDS, NIAID, NIH: 447-52D and 240-D from Dr. Susan Zolla-Pazner, D5 from Dr. Danilo Casimiro, F425-B4e8 and F105 from Dr. Marshal Posner and Dr. Lisa Cavacini, #564 from Dr. Michael H. Malim, 1G7 from Dr. Anne Marie Szilvay, and 1D9 from Dr. Dag E. Helland. Binding epitopes of all HIV-1 specific mAbs are indicated in S1 Fig.

HIV-1 gp41 protein [21, 22] as well as peptides 94.1 [23], bio-57, bio-0705 [24], and IZM were kindly donated by Michael B. Zwick and Philip Dawson. MPER peptide C22-pT [25] was generously provided by Min Lu. Recombinant HIV-1 gp140 (UG37, SF162 [26, 27] (Dr. Leo Stamatatos), CN54, UG21, BR29) and HIV-1 gp120 (BaL, CN54 [28, 29] (Dr. Ian Jones), 96ZM651, 93TH975) envelope proteins were also obtained through the NIH AIDS Reagent Program, Division of AIDS, NIAID, NIH. Clade specificity and expression hosts of all recombinant HIV-1 envelope proteins used in this study are shown in S1 Table.

### Peptide Synthesis

The MPER analogs 09129, 09122, 09128, and 08023 (Table 1) were manually synthesized, using butoxycarbonyl solid phase synthesis, as described previously by Ingale and colleagues [24]. Following synthesis, the peptides were cleaved from the resin with hydrogen fluoride, purified by HPLC, and analyzed by mass spectrometry. In contrast, trimeric MPER peptide C22-pT was originally expressed in *E*. *coli* as inclusion bodies, purified, and cleaved with cyanogen bromide as reported elsewhere [25].

**Table 1:** Sequence alignment of MPER peptides printed on the microarray chip and used for in solution competition assay with HIV-1_JR-FL_ MPER.

Analog	Sequence	References
MPER_JR-FL_	_659_ELL**ELDKWA**SLWN**WFDITN**WLWYIK_683_	
09129	LLELDKWASLWNWFDITNWLWYIKKKK-NH_2_	[52]
09122	SLWNWFDITNWLWYIKKKK-NH_2_	[24]
09128	ELDKWASLWNWF**D**ITNWLWYIK(IEELKSKIKRIENEIKRIKK)_3_	-
C22-pT	ELDKWASLWNW**F**NITNWLWYIK(IEELKSKIKRIENEIKRIKK)_3_	[25]
IZM	(IEELKSKIKRIENEIKRIKK)_3_	-
94.1	NWFDITNWLWYIKKKK-NH_2_	-
bio-0705	NWFDITNWLWYIKKKK-bio-NH_2_	-
bio-57	ELLELDKWASLWNRRK-bio-NH_2_	-
08023	WTLNIFWIWSYLDWSNKKKK-NH_2_	-

Numbering according to clade B isolate HIV-1_HxB2_. MPER analogs 09128 and C22-pT are both trimerized through an isoleucine zipper motif at the C-terminus. The amino acid position at 674 of both peptides is indicated in bold. Analog 08023 is a scrambled negative control peptide based on MPER sequence of analog 09122. The epitope for 2F5 (ELDKWA) and 4E10 (WFDITN) is indicated in bold.

### Microarray fabrication and hybridization

For microarrays, all HIV-1 envelope proteins and MPER analogues were printed at three different concentrations (1.0 mg/mL, 0.1 mg/mL, and 0.01 mg/mL) onto nitrocellulose coated glass FAST slides (Whatman), using an Omni Grid 100 microarray printer (Genomic Solutions). This corresponds to a total protein amount of 1.0 ng, 0.1 ng, and 0.01 ng per spot, respectively. For array probing, HIV-1 specific mAbs and plasma samples of HIV-1 infected subjects were diluted in Protein Array Blocking Buffer (Whatman, GE Healthcare), supplemented with 10% (vol/vol) DH5α *E*. *coli* lysate and incubated on arrays over-night (o/n) at 4°C with constant agitation. *E*. *coli* lysate is routinely used in our assays to remove background binding especially when working with serum samples. Antibody concentrations for the probing ranged from 10 μg/mL to 0.003 μg/mL using serial half-log dilution steps. Plasma samples were probed at a dilution of 1:100.

Following o/n incubation, slides were washed five times in washing buffer (10 mM Tris (pH 8.0), 150 mM NaCl), containing 0.05% Tween 20, and bound human antibodies were detected by biotin SP-conjugated affini-pure goat anti-human IgG Fc fragment specific secondary antibody (Jackson ImmunoResearch, West Grove, PA), diluted 1/200 in blocking buffer. After 1 h at room temperature (RT), slides were washed three times, and bound antibodies were detected with streptavidin-conjugated SureLight P-3 tertiary reagent (Columbia Biosciences, Columbia, MD), diluted 1/200 in blocking buffer, for 1 h at RT. Slides were then washed 5 times, air dried under brief centrifugation, and stored at 18°C in a desiccator. The arrays were examined with a Perkin Elmer ScanArray Express HT confocal laser scanner at a wavelength of 670 nm and signal intensities were quantified using ProScanArray Express software (Perkin Elmer, Waltham, MA). All signal intensities were corrected for spot-specific background. Only signal intensities above a certain threshold signal (i.e. higher than controls) were considered positive, and used for the calculation of median signal intensities or percentages of bound proteins. Information on the microarray platform is publicly available on NCBI’s Gene Expression Omnibus and is accessible through GEO Platform accession number GSE66659.

### Specificity ELISA

96-well plates (Costar) were coated with 2 μg/mL (100 ng per well) of respective antigen (gp140, gp120, gp41 or MPER peptide) diluted in PBS and incubated o/n at 4°C. Plates were washed, blocked with 4% non-fat dry milk (NFDM) in PBS containing 0.05% Tween 20, and incubated for 1 h at RT. Serial dilutions (1:4) of mAbs (starting concentration of 10 μg/mL) in 1% NFDM were prepared and added to the washed plates. After 1 h at RT unbound antibodies were removed and bound antibodies were detected by a HRP-labeled goat anti-human Fc gamma specific conjugate (Sigma-Aldrich). Plates were finally washed, developed with TMB (3,3′,5,5′-tetramethylbenzidine) substrate, stopped with 2M H_2_SO_4_, and read on a BioTek plate reader at a wavelength of 450 nm. All assays were performed at least twice. Half maximal effective concentration (EC_50_) was calculated using GraphPad PRISM 5.0 software.

### In-solution competition ELISA

The in-solution competition ELISA was performed according to Stanfield and colleagues [30]. In brief, 96-well plates were coated o/n with 200 ng/well neutravidin (Pierce) in PBS at 4°C and blocked with 4% NFDM for 1 h at RT. Different competing MPER peptide analogs (serially diluted 1:3 in 1% NFDM at a starting concentration of 0.04 mM) were mixed with a constant concentration of biotinylated peptide bio-0705 (0.1 μg/ml) or bio-57 (0.1 μg/ml) and the corresponding human mAb 4E10 (0.5 μg/ml) or mAb 2F5 (0.2 μg/ml), respectively. The antibody/peptide mixture was incubated for 2 h at 37°C, subsequently transferred to the neutravidin plates and incubated at 37°C for 20 min. Bound antibodies were detected with HRP labeled goat anti human F(ab)_2_ conjugate (Pierce). After 1 h at RT, wells were developed, stopped and read as described above. The concentration of competitor peptide corresponding to a half-maximal signal (IC_50_) was determined by nonlinear fit interpolation of the resulting binding curve (GraphPad PRISM 5.0 software). Each peptide competitor was tested in duplicate in at least two separate experiments.

### Virus generation and neutralization assay

Pseudotyped virions were generated by co-transfecting 5 x 10^5^ 293T cells with a mixture of pSG3ΔEnv backbone plasmid (4 μg), HIV-1 JR-FL, HIV-1 JR-FL_D674N_, JR-2 or JR-2_D674N_ envelope complementation plasmid (2 μg), and polyethyleneimine (PEI) at a DNA/PEI ratio of 1/3. Two days post transfection, virus containing supernatant was harvested and cleared by centrifugation at 4000 rpm for 10 min. TZM-bl single round infectivity assay was performed as described elsewhere [31]. Briefly, pseudotyped virus was added at a 1:1 ratio to serially diluted (1:3) mAbs (starting at 100 μg/mL) and incubated at 37°C. After 1 h, TZM-bl reporter cells were added (1:1 by volume) at 1×10^4^ cells/well in a final concentration 10 μg/mL DEAE-dextran, and incubated for 48 h at 37°C. Plates were processed, and developed with luciferase assay reagent, according to the manufacturer’s instructions (Promega). Luminescence in relative light units (RLUs) was measured using a microplate luminometer (BioTek). All experiments were performed at least in duplicate. The extent of virus neutralization in the presence of antibody was determined at the 50% inhibitory concentration (IC_50_) in the absence of mAb.

## Results

### Antibody probing reveals diverse binding patterns against HIV-1 specific antigens

**Fig 1:**
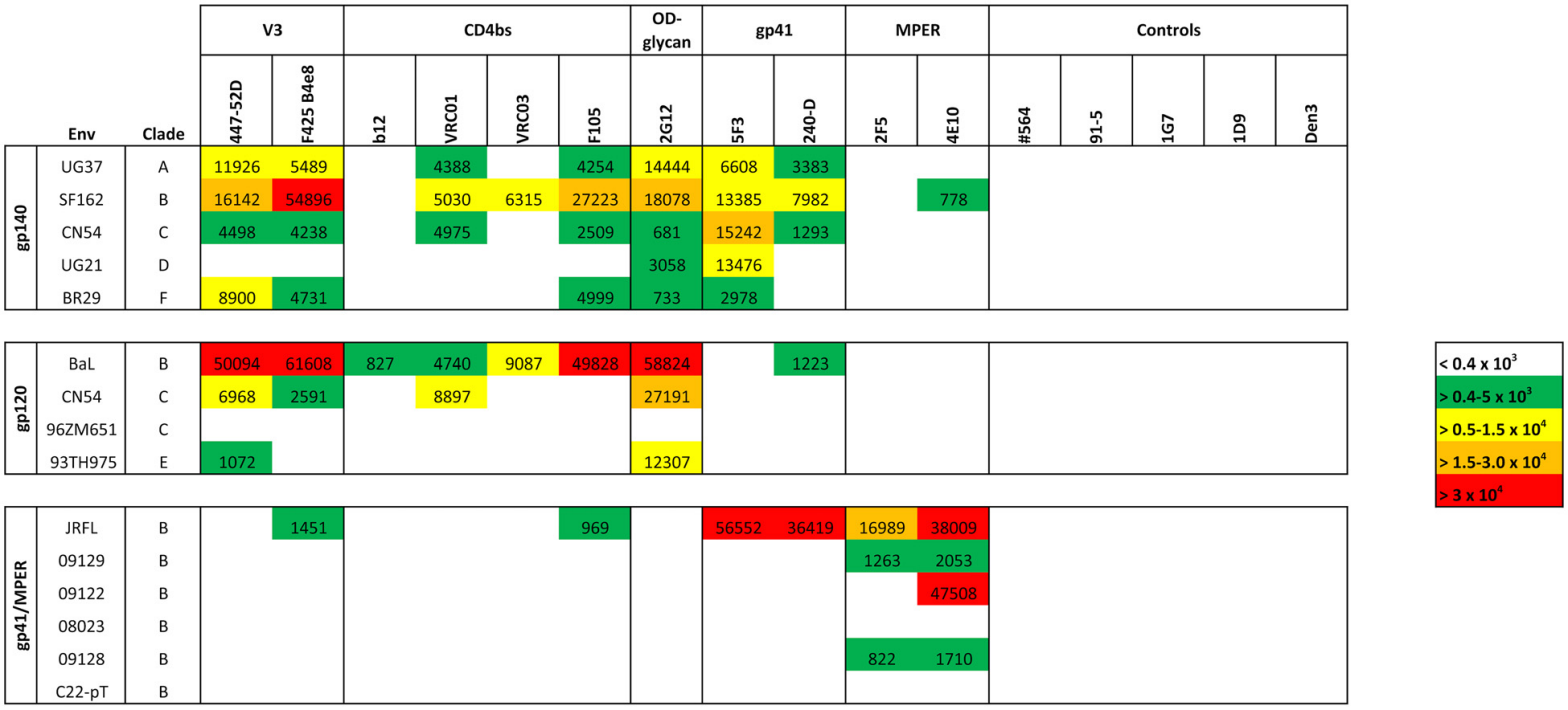
HIV-1 protein microarray chip analysis. HIV-1 specific antibodies were tested against a panel of recombinant multi-clade gp140 and gp120 proteins as well as gp41 and MPER analogs. Proteins were printed at a concentration of 0.1 mg/mL, which corresponds to 0.1 ng per spot. Antibodies were probed at a concentration of 10 μg/mL. The cutoff for positive binding was a signal intensity of 0.4 x 10^3^. Signal intensities were color-coded using green (>0.4–5.0 x 10^3^) for weak, yellow (>0.5–1.5 x 10^4^) for intermediate, orange (>1.5–3.0 x 10^4^) and red (>3.0 x 10^4^) for strong interaction. Non-specific binding was indicated as white (<0.4 x 10^3^) boxes. Data are representative of at least two independent experimental runs.

To examine binding attributes of multiple HIV-1 envelope specific antibodies simultaneously, a total of 15 HIV-1 multi-clade envelope proteins, including five gp140, four gp120, one gp41 protein, and six gp41 MPER analogs, were spotted onto microarray chips. All MPER peptides, 09129, 09122, 08023, 09128, and C22-pT (Table 1), were analyzed for mAb 2F5 and mAb 4E10 binding by an in-solution competition assay prior to printing on the protein microarray chip. Analog 09128, a modified version of MPER analog C22-pT, showed remarkably enhanced reactivity to both mAbs 2F5 (IC_50_ of 76.3 nM) and 4E10 (IC_50_ of 0.9 nM) in comparison to wild type protein C22-pT (2F5 and 4E10, IC_50_ of >10,000 nM and 3269 nM, respectively), indicating a positive effect of the introduced N674D mutation (S2 Fig). As expected, full-length MPER peptide 09129 was recognized by both MPER antibodies 2F5 (IC_50_ of 2031 nM) and 4E10 (IC_50_ of 1070 nM), whereas peptide 09122 interacted only with 4E10 (IC_50_ 115 nM). In contrast, a sequence scrambled control MPER analog 08023, designed after the peptide sequence of 09122, did not interact with any of the tested antibodies (S2 Fig). Based on their reactivity profiles, MPER analogs 09128, C22-pT, 09129, 09122, and 08023 were subsequently used in the chip analysis to screen for 2F5- and 4E10-like antibodies. Each protein and peptide was printed in three concentrations: 1.0 mg/mL, 0.1 mg/mL, and 0.01 mg/mL, corresponding to 1.0 ng, 0.1 ng, and 0.01 ng per spot. After antibody probing and signal quantification, antigens were sorted by envelope and clade into three major clusters (gp140, gp120, and gp41/MPER). Additionally, probed mAbs were grouped into six categories based on their epitope specificity: 1) variable loop 3 (V3) antibodies, 2) CD4bs antibodies, 3) glycan-specific antibodies 4) gp41 antibodies, 5) MPER specific antibodies, and 6) control antibodies. For the generation of the heat map in Fig 1, only antibody signals at non-saturating antigen concentrations were considered in order to decrease nonspecific antibody binding due to high protein concentrations. The optimal HIV-1 protein concentration for mAbs was 0.1 mg/mL, or 0.1 ng per spot. The heat map in Fig 1 shows signal intensities that reveal a diverse binding pattern for each antibody class across the tested antigens.

### Variable loop specific antibodies

**Fig 2:**
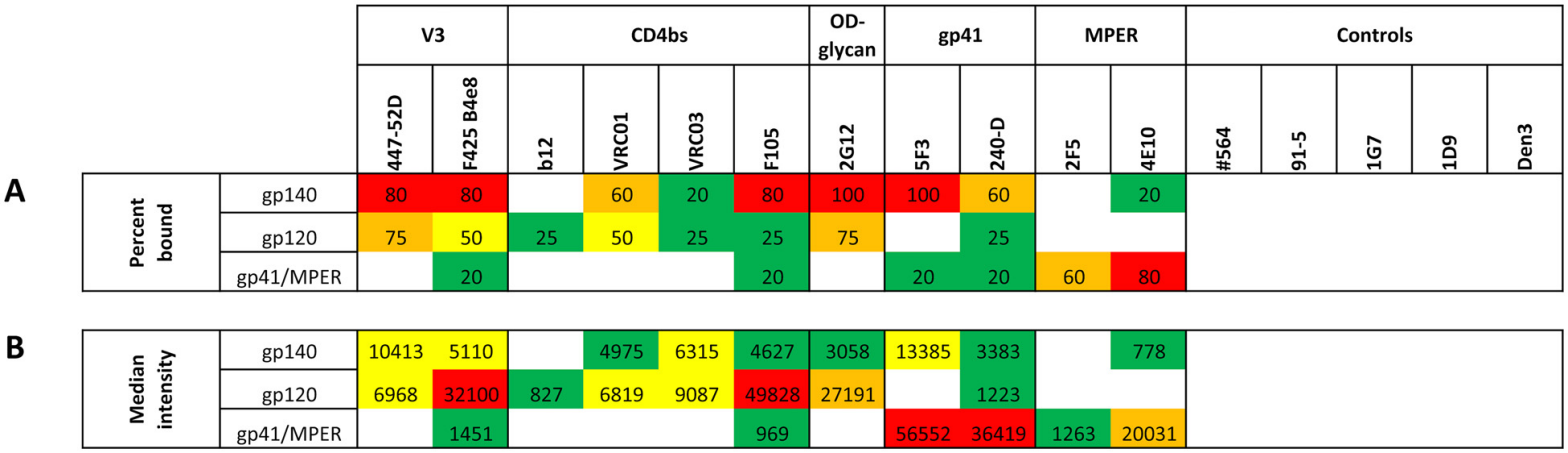
Binding breadth and median signal intensities of HIV-1 specific antibodies. (A) The binding breadth is indicated by the percentage of bound proteins and color-coded as follows white (<0%), green (>0–25%), yellow (>25–50), >50–75% (orange), and >75% (red). The sample size was N = 5 for the gp140 cluster, N = 4 for the gp120 cluster, and N = 5 for the gp41/MPER cluster, as the peptide 08023 was excluded. (B) Median signal intensities of bound antibodies were calculated based on signal intensities derived from Fig 1, and plotted into a signal heat map with the same color code as in Fig 1, using green (>0.4–5.0 x 10^3^) for weak, yellow (>0.5–1.5 x 10^4^) for intermediate, orange (>1.5–3.0 x 10^4^) and red (>3.0 x 10^4^) for strong interaction. Non-specific binding was indicated as white (<0.4 x 10^3^) boxes. The cutoff for positive binding was a signal intensity of 0.4 x 10^3^.

To compare antibodies within each subclass, percentages and median signal intensities of bound envelope proteins were calculated, and plotted in Fig 2. Percentages of bound envelope proteins were calculated based on a sample size (N) of N = 5 for the gp140 cluster, N = 4 for the gp120 cluster, and N = 5 for the gp41/MPER cluster. The scrambled control peptide 08023 of the gp41/MPER cluster was excluded from the calculation. As indicated in Fig 2A and 2B, the V3 loop-specific mAbs 447-52D and F425 B4e8 exhibited strong cross clade coverage, and intermediate signal intensities throughout the gp140 cluster and gp120 cluster. Highest signal intensities for both antibodies were observed with clade B HIV-1_BaL_ gp120 and clade B HIV-1_SF162_ gp140, followed by intermediate clade A HIV-1_UG21_ gp140 and clade F HIV-1_BR29_ gp140 binding in case of mAb 447-52D (Fig 1), suggesting a predominant clade B specificity of both V3-loop specific antibodies. In contrast, intermediate to weak binding was observed against HIV-1_CN54_ gp140 and HIV-1_CN54_ gp120, whereas very weak binding was detected against HIV-1_93TH975_ gp120 (447-52D). None of the V3 loop-specific mAbs reacted against clade D HIV-1_UG21_ gp140 and clade C HIV-1_96ZM651_ gp120. The strong cross clade reactivity of mAbs 447-52D and F425 B4e8 is consistent with the moderate neutralization breadth observed against HIV-1 primary isolates. Weak poly-reactivity (i.e. signal intensity higher than background level) against HIV-1_JRFL_ gp41 in the gp41/MPER cluster was observed with mAb F425 B4e8. Some of the poly-reactivity observed might be due to the distinct binding mechanism of mAb F425 B4e8, compared to other V3-specific antibodies like 447-52D. Contact between mAb F425 B4e8 and its linear epitope in the tip of the V3 loop is mainly made through side-chain contacts involving only two residues, thus explaining the considerable cross clade reactivity of this antibody [32].

### CD4bs specific antibodies

The CD4bs specific antibodies F105, VRC01, and VRC03 revealed high to weak reactivity across the gp140 cluster and the gp120 cluster at a protein concentration of 0.1 ng/spot (Fig 2A). Broadly neutralizing mAbs VRC01 and VRC03 exhibited intermediate to weak median cross clade signal intensities against the gp140 and gp120 cluster (Fig 2B). In contrast, weakly neutralizing mAb F105 showed a strong median cross clade signal intensity against the gp120 cluster, followed by weak binding against the gp140 cluster. Best binding of mAbs VRC01, VRC03, and F105 was achieved against clade B HIV-1_SF162_ gp140, and clade B HIV-1_BaL_ gp120 as well as clade C HIV-1_CN54_ gp120 in case of VRC01 (Fig 1). The broadly neutralizing mAb b12 interacted predominantly with clade B in the gp120 cluster. However, the interaction with HIV-1_BaL_ gp120 was rather weak (Fig 1). Weak poly-reactivity against HIV-1_JRFL_ gp41, similar to F425 B4e8, was observed with mAb F105.

### Glycan specific mAb 2G12

MAb 2G12, the only glycan specific mAb analyzed in this panel, was highly cross-reactive against all tested gp140 and gp120 glycoproteins (Fig 2A). Highest median signal intensities were found against the gp120 cluster (Fig 2B). As indicated in Fig 1, mAb 2G12 revealed strong binding against clade B HIV-1_BaL_ gp120 and HIV-1_SF162_ gp140, clade C HIV-1_CN54_ gp120, and intermediate binding signals against clade A HIV-1_UG37_ gp140 and clade E HIV-1_93TH975_ gp120. The signal intensities of mAb 2G12 in the gp140 cluster are in good agreement with a recent study where broadly neutralizing mAb 2G12 was able to neutralize isolates from various clades with varying efficiency [33, 34]. Minimal reactivity of mAb 2G12 was found against HIV-1_JRFL_ gp41. However, the signal was below our signal intensity cutoff level of 0.4 x 10^3^, and only visible at the highest antibody concentration (10 μg/mL) tested.

### Gp41 specific antibodies

The gp41-specific mAb 5F3 (epitope: ^650^QNQQEKNE^657^, cluster II) showed high reactivity across the gp140 cluster (Fig 2A), with strong to intermediate signal intensities against clades A to D (Fig 1). The signal intensity of mAb 5F3 against clade F HIV-1_BR29_ gp140 was about 2 to 6-fold lower than against the other clades (Fig 1). In contrast, cluster I specific mAb 240-D (epitope: ^592^LLGIWGCSGKLICTT^606^) reacted predominantly with clade B HIV-1_SF162_ gp140, followed by clade A HIV-1_UG37_ gp140, and clade C HIV-1_CN54_ gp140. Interestingly, mAb 240-D revealed poly-reactivity against HIV-1_BaL_ gp120 (Fig 1). Although, poly-reactivity is common phenomenon amongst cluster II gp41 antibodies it has not been found with cluster I specific antibodies such as 240-D [35]. Noteworthy, although mAb 5F3 belongs to the class of non-neutralizing antibodies, it exhibits remarkable cross clade reactivity. The epitope amino acid sequence numbering follows HIV-1_HXB2_ gp160 (HIV databases: http://www.hiv.lanl.gov).

### MPER specific antibodies

As shown in Fig 1, MPER antibody 4E10 reacted only minimally with HIV-1_SF162_ gp140 in the gp140 cluster, and as expected no signal intensities were observed against the gp120 cluster (Fig 1). In contrast, mAb 2F5 did not interact with envelopes of the gp140 or the gp120 cluster. All tested MPER antibodies strongly interacted with HIV-1_JRFL_ gp41. Weak binding was observed against full-length MPER peptide 09129, and trimeric full-length MPER peptide 09128. Additionally, mAb 4E10 revealed strong signal intensity against MPER analog 09122. No binding was found against control peptide 08023 and C22-pT, which is in good agreement with the in solution capture assay shown in S2 Fig.

### Control antibodies

Control antibodies against non-envelope HIV-1 proteins Vif (#564), p24 (#91–5), Rev (1G7), and Tat (1D9) as well as the non HIV-1 specific mAb Den3 exhibited no reactivity against all tested proteins, indicating overall low vulnerability of the platform for unspecific binding.

### Cluster analysis of antibody specificity against gp140 and gp41 constructs corroborates distinct binding patterns

**Fig 3:**
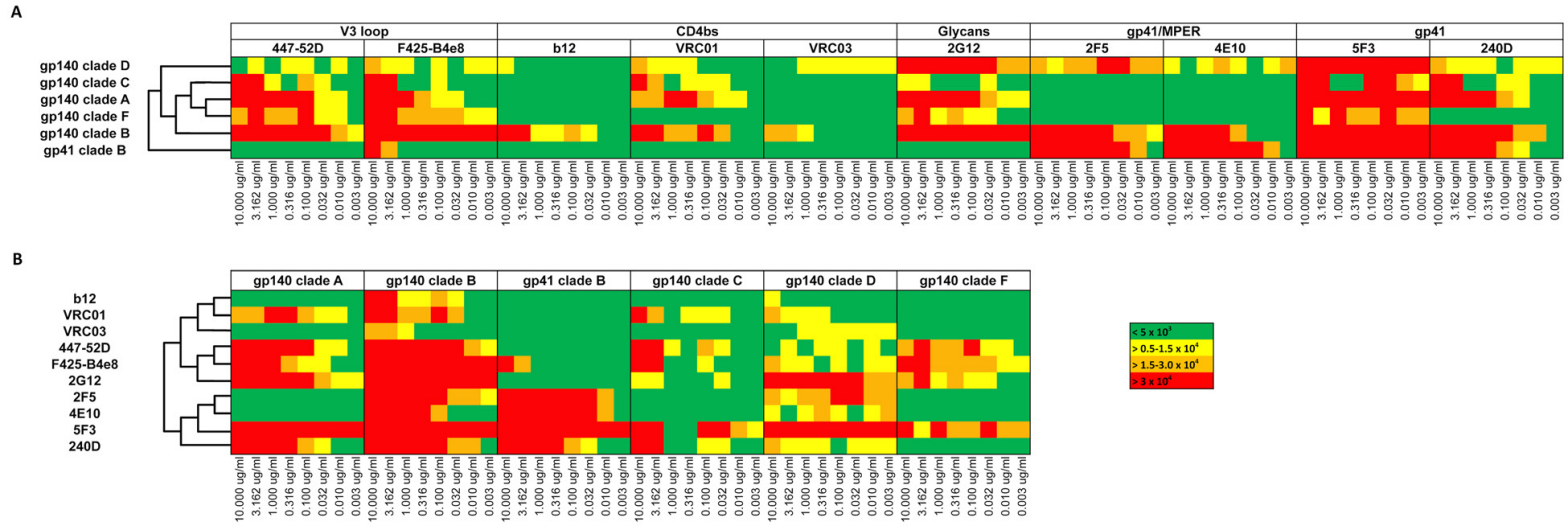
Cluster analysis. Arrays were probed with mAbs diluted in a series of 8 half log concentrations as described in the text. The heat map displays intensity with orange (>1.5–3.0 x 10^4^) and red (>3.0 x 10^4^) showing the strongest, green (<5.0 x 10^3^) the weakest, and yellow (>0.5–1.5 x 10^4^) intermediate according to Fig 1. MeV v4.6 (TM4Microarray Software Suite, www.tm4.org) was used to perform the hierarchical clustering analysis using average linkage clustering. Samples are clustered according to response against protein antigen spots (1.0 ng per spot) on the array.

To evaluate the microarray chip for specific antibody binding pattern, a hierarchical cluster analysis was performed. As shown in Fig 3, reactivity of 10 mAbs against 6 HIV antigens consisting of five multi-clade (A, B, C, D, F) gp140 molecules and a clade B gp41 molecule was compared and plotted into a heat map. Antibodies were probed on separate arrays using 8 serial half-log dilutions ranging from 10 μg/mL to 0.003 μg/mL for each mAb. To evaluate antibody-binding patterns, the highest antigen concentration of 1.0 mg/mL (1.0 ng per spot) was used. Although, we observed unspecific binding for some of the antibodies (e.g. F425 B4e8 against gp41_JRFL_) at the highest concentrations we found that binding does not impact the analysis, as the binding patterns of the lower antibody concentrations contribute the most. The results are summarized on the heat maps in Fig 3. Unbiased hierarchical clustering revealed a distinct pattern of reactivity for each of the 6 antigens (Fig 3A and 3B). The anti-gp41 antibody 5F3 was reactive against all tested antigens, followed by 240-D which exhibited a strong reactivity against clade A, B, and C constructs. In contrast, MPER specific mAbs, 4E10 and 2F5 reacted preferentially with gp41 and gp140 from clade B, and gp140 from clade D. Out of the six gp120 specific antibodies tested, the two V3 loop specific mAbs 447-52D and F425-B4e8 showed high cross clade reactivity, followed by glycan specific mAb 2G12, and CD4bs specific mAb VRC01. In sharp contrast, CD4bs antibodies b12 and VRC03 reacted predominantly with clade B. The reactivity of the mAbs was also clustered into distinct groups according to their published classification (Fig 3B). The CD4bs mAbs (VRC01, VRC03 and b12) clustered together and the V3 loop specific mAbs (447-52D and F425-B4e8) formed their own distinct cluster. The glycan specific mAb 2G12 came out as a singleton, whereas the gp41 specific mAbs clustered together with the MPER specific mAbs forming a distinct pair. This result is in good agreement with the published classification of each antibody and illustrates how chip-binding data, followed by hierarchical cluster analysis, may be applied for the identification and classification of unknown antibody specificities.

### Microarray binding profile of mAbs correlate significantly with binding profile from ELISA experiments

**Fig 4:**
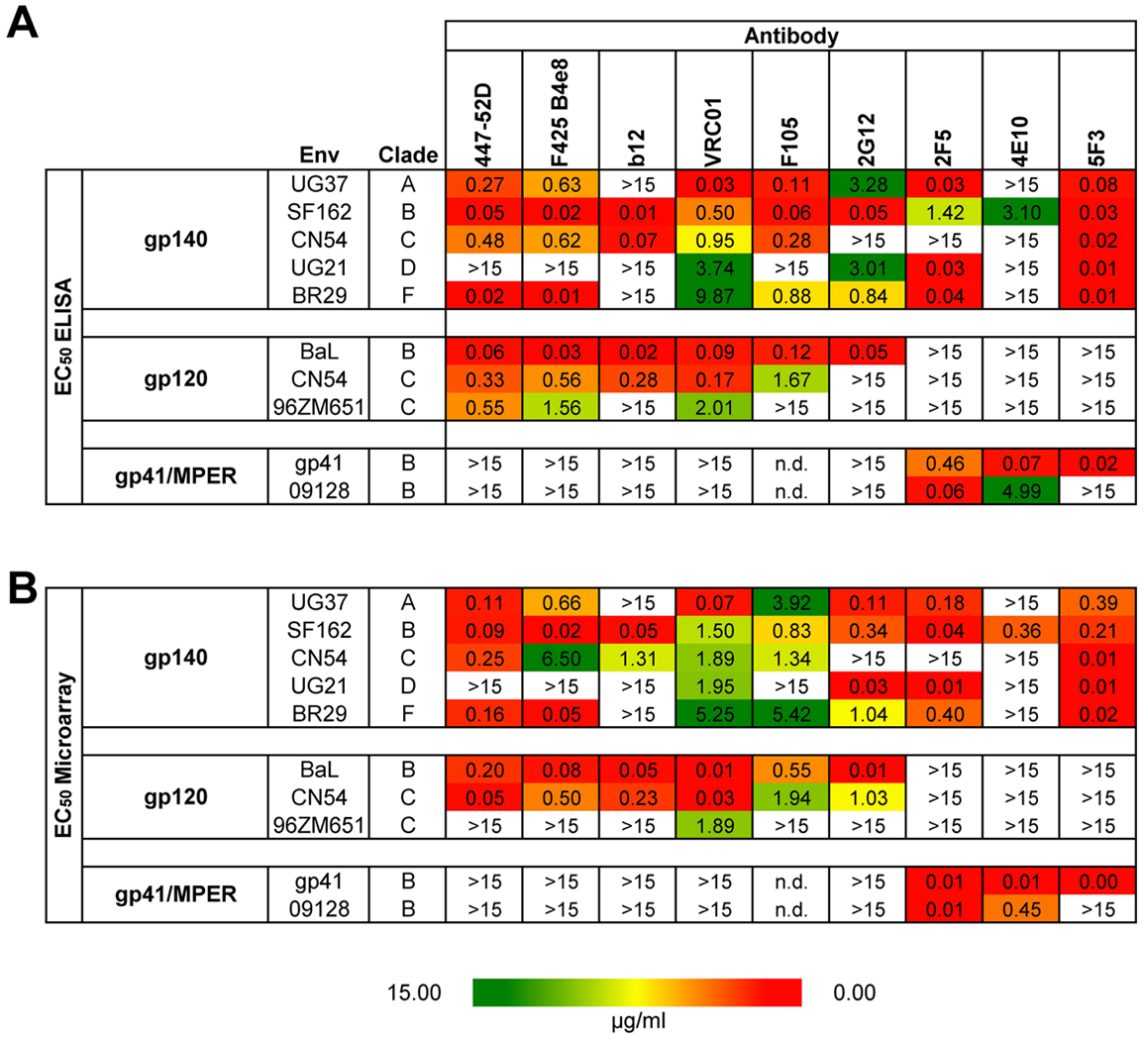
Calculation and evaluation of EC_50_ data. EC_50_ values (μg/mL) were generated and calculated (PRISM GraphPad 5.0) by antibody binding curves in ELISA (A) and in the protein microarray chip assay (B). Values were color-coded using red for low, yellow for medium, and green for high EC_50_ values. No binding is indicated as white boxes. EC_50_ values of both analyses are representative of two independent experiments.

To confirm the binding data obtained from protein microarray analysis, mAbs were further evaluated by serial dilutions, both on the chip as well as in a conventional ELISA setup. EC_50_ values were calculated from both platforms and plotted into a 3-color scale heat map. As indicated in Fig 4, binding patterns were remarkably consistent. Next, EC_50_ values from each group were further evaluated for significant correlations. Spearman’s rank correlation coefficient (ρ) was calculated using GraphPad PRISM 5.0. As indicated in S2 Fig, the two groups strongly correlated with a ρ value of 0.8839 and a P value of <0.0001 validating the microarray as an alternative method to conventional ELISA.

### Equilibrium dissociation constant (Kd) calculation reveals various binding affinities towards tested antigens

To further define binding affinities, Kd’s of antibody-antigen interaction from binding curves (S3 Fig) were calculated, using a one-site binding algorithm (GraphPad PRISM 5.0). For each mAb, the corrected Bmax (Bmax/mg antigen printed) and the affinity constant (Ka) (Table 2) were determined (1/Kd). All three classes of gp120 specific antibodies displayed varying binding affinities towards gp140 antigens while showing no detectable binding to HIV-1_JRFL_ gp41 (Table 2). Besides the lack of binding to gp41, the CD4bs antibodies b12 and VRC03 showed low or no binding affinities towards the gp140 antigens printed, with the exception of HIV-1_SF162_ gp140 for which they exhibited weak affinity (Table 2). The glycan specific mAb 2G12 exhibited intermediate binding affinities toward all gp140 antigens except gp41. On the contrary, the gp41 and MPER specific mAbs analyzed revealed high affinities for both gp41 and gp140 antigens from clades A, B, C, and D, but did not show any binding affinity against HIV-1_BR29_ gp140 from clade F (Table 2).

**Table 2:** Antibody affinity calculations.

	Antibody	Env	Env conc.	Clade	cBmax	KA (nM)
**gp120 V3 loop specific**	**447-52D**	UG37 gp140	1.0 mg/mL	A	85063	1.161
		SF162 gp140	0.1 mg/mL	B	258070	0.406
		CN54 gp140	1.0 mg/mL	C	107019	0.039
		UG21 gp140	-	D	n.b.	n.b
		BR29 gp140	1.0 mg/mL	F	120280	0.467
		JRFL gp41	-	B	n.b.	n.b
	**F245—B4e8**	UG37 gp140	1.0 mg/mL	A	85436	0.247
		SF162 gp140	0.1 mg/mL	B	537320	4.112
		CN54 gp140	1.0 mg/mL	C	138639	0.013
		UG21 gp140	1.0 mg/mL	D	34827	0.020
		BR29 gp140	1.0 mg/mL	F	233027	2.109
		JRFL gp41	1.0 mg/mL	B	401471	0.474
**gp120 CD4bs specific**	**b12**	UG37 gp140	-	A	n.b.	n.b
		SF162 gp140	1.0 mg/mL	B	66410	0.037
		CN54 gp140	-	C	n.b.	n.b
		UG21 gp140	-	D	n.b.	n.b
		BR29 gp140	-	F	n.b.	n.b
		JRFL gp41	-	B	n.b.	n.b
	**VRC01**	UG37 gp140	1.0 mg/mL	A	35224	2.898
		SF162 gp140	1.0 mg/mL	B	70159	0.137
		CN54 gp140	1.0 mg/mL	C	44444	0.181
		UG21 gp140	1.0 mg/mL	D	15161	0.285
		BR29 gp140	-	F	n.b.	n.b
		JRFL gp41	-	B	n.b.	n.b
	**VRC03**	UG37 gp140	-	A	n.b.	n.b
		SF162 gp140	1.0 mg/mL	B	19059	0.202
		CN54 gp140	-	C	n.b.	n.b
		UG21 gp140	-	D	n.b.	n.b
		BR29 gp140	-	F	n.b.	n.b
		JRFL gp41	-	B	n.b.	n.b
	**F105**	UG37 gp140	1.0 mg/mL	A	90493	0.028
		SF162 gp140	1.0 mg/mL	B	66364	0.115
		CN54 gp140	1.0 mg/mL	C	26168	0.117
		UG21 gp140	1.0 mg/mL	D	19421	0.036
		BR29 gp140	1.0 mg/mL	F	254253	0.010
		JRFL gp41	1.0 mg/mL	B	476630	0.001
**gp120 glycan dependent**	**2G12**	UG37 gp140	1.0 mg/mL	A	81548	2.500
		SF162 gp140	0.1 mg/mL	B	200260	0.798
		CN54 gp140	1.0 mg/mL	C	13983	0.279
		UG21 gp140	0.1 mg/mL	D	41660	0.441
		BR29 gp140	1.0 mg/mL	F	98489	0.713
		JRFL gp41	-	B	n.b.	n.b
**gp41 specific**	**5F3**	UG37 gp140	0.1 mg/mL	A	47600	0.099
		SF162 gp140	0.1 mg/mL	B	393930	199.960
		CN54 gp140	0.1 mg/mL	C	251338	1.330
		UG21 gp140	0.1 mg/mL	D	294750	80.906
		BR29 gp140	-	F	n.b.	n.b
		JRFL gp41	0.1 mg/mL	B	612410	391.696
	**240-D**	UG37 gp140	1.0 mg/mL	A	77744	15.076
		SF162 gp140	0.1 mg/mL	B	65685	66.534
		CN54 gp140	0.1 mg/mL	C	51653	16.559
		UG21 gp140	0.1 mg/mL	D	16682	1.688
		BR29 gp140	-	F	n.b.	n.b
		JRFL gp41	1.0 mg/mL	B	67843	12.832
**MPER specific**	**2F5**	UG37 gp140	-	A	n.b.	n.b
		SF162 gp140	1.0 mg/mL	B	62458	4.728
		CN54 gp140	-	C	n.b.	n.b
		UG21 gp140	-	D	n.b.	n.b
		BR29 gp140	-	F	n.b.	n.b
		JRFL gp41	0.1 mg/mL	B	51640	10.000
	**4E10**	UG37 gp140	-	A	n.b.	n.b
		SF162 gp140	1.0 mg/mL	B	64014	0.697
		CN54 gp140	-	C	n.b.	n.b
		UG21 gp140	1.0 mg/mL	D	11503	1.795
		BR29 gp140	-	F	n.b.	n.b
		JRFL gp41	1.0 mg/mL	B	59917	5.580

Corrected Bmax and affinity constant (Ka) values for the interaction between HIV-1 envelope-specific mAbs and HIV-1 envelope glycoproteins from clades A, B, C, D, and F were determined by GraphPad PRISM 5.0. Corrected Bmax (cBmax) and Ka values are representative of at least two independent experiments.

### Probing the chip with HIV-1 infected human plasma yields patient specific binding patterns

**Fig 5:**
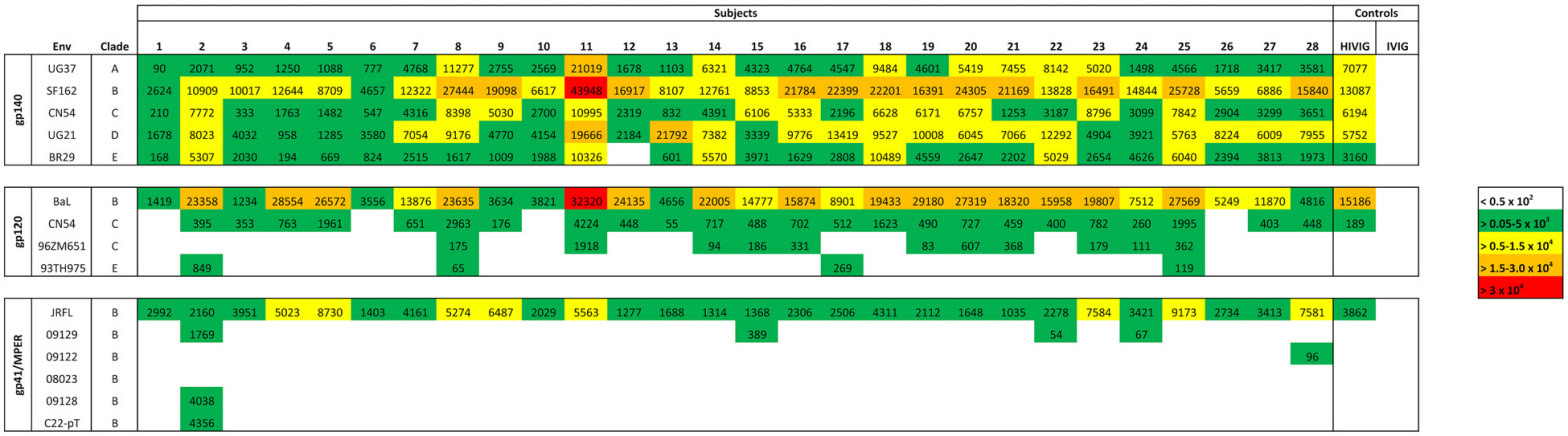
Microarray analysis of HIV-1 infected patient samples. The microarray chip was probed with HIV-1 patient plasma at a dilution factor of 1:100. Proteins were printed at a concentration of 0.01 mg/mL, which corresponds to 0,01 ng per spot. HIVIG and IVIG were included as a positive control and negative control, respectively. Intensities were color coded using green (>0.05–5.0 x 10^3^) for weak, yellow (>0.5–1.5 x 10^4^) for intermediate, orange (>1.5–3.0 x 10^4^) and red (>3.0 x 10^4^) for strong interaction. Non-specific binding was indicated as white (<0.5 x 10^2^) boxes. Data are representative of at least two independent experimental runs.

**Fig 6:**
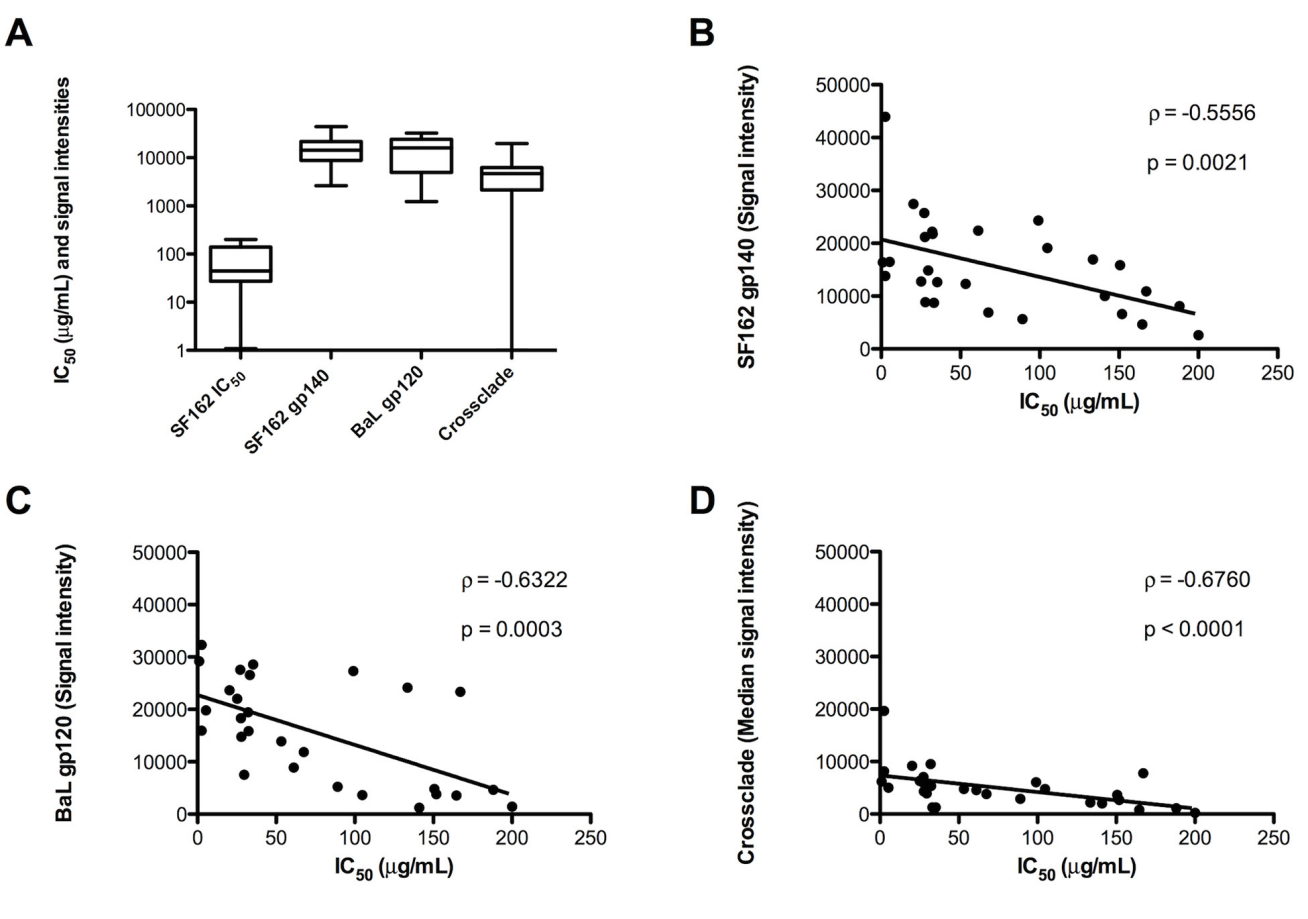
Patient plasma sample evaluation. Plasma samples of HIV-1 infected subjects were analyzed for neutralization capacity against Tier 1 isolate HIV-1_SF162_ next to median binding intensities against HIV-1_SF162_ gp140, HIV-1_BaL_ gp120 and cross clade signal intensity within the gp140 cluster (A). Further, correlations were calculated between IC_50_ values and 1) median HIV-1_SF162_ gp140 signal intensities (B), 2) median HIV-1_BaL_ gp120 signal intensities (C), and 3) cross clade gp140 signal intensities (D).

We probed and analyzed plasma samples from 28 subjects with chronic HIV-1 infection on suppressive ART to investigate potential clinical applications of the microarray chip. Plasma samples were diluted 1/100 prior to probing. The optimal antigen concentration used for statistical analysis and heat map generation was 0.01 mg/mL or 0.01 ng per spot. As shown in Fig 5, plasma from HIV infected subjects demonstrated different antibody binding intensities across the tested envelope proteins. Twelve subjects revealed intermediate median signal intensities throughout the gp140 cluster, thus indicating a high cross-clade specificity for these antigens. This trend was similar to the positive control HIVIG (pool of purified HIV-1 infected donor IgG). Four additional subjects (7, 9, 15, and 17) showed marginally lower median signal intensities. The remaining twelve subjects revealed substantially lower (weak) median signal intensities. Based on the median signal intensities within each subtype of the gp140 cluster, patients were predominantly reactive to clade B followed by clades D, A, C, and F. A similar trend in reactivity was found for the gp120 cluster where subjects mainly reacted with clade B followed by clade C (HIV-1_CN54_ gp120). Minimal reactivity was observed with HIV-1_96ZM651_ gp120 and HIV-1_93TH975_ gp120. Binding pattern data of the chip analysis provide clear support for the notion that most of the subjects were originally infected with clade B HIV-1. All patients showed intermediate to weak reactivity against HIV-1_JRFL_ gp41 suggesting utmost specificity at the tested concentration. Additional binding was found against MPER peptide 09129 and 09122, although at rather weak signal intensities. Only subject 2 recognized peptides C22-pT and 09128. None of the subjects reacted with the scrambled control peptide 08023 indicating that antibody response against the other MPER analogs is highly specific. The polyclonal antibody control IVIG (pool of purified IgG from healthy donors) revealed minor antigen interaction within the gp120 cluster. The signal intensities of IVIG served as a cutoff level for positive binding of plasma samples (Fig 5). Patient sera were evaluated for their ability to neutralize a relatively sensitive Tier 1 isolate HIV-1_SF162_ (Fig 6A). When we correlated the binding pattern of HIV-1_SF162_ gp140 and HIV-1_BaL_ gp120 we found a significant correlation between antibody binding signals and IC_50_ values (HIV-1_SF162_ gp140 and HIV-1_SF162;_ P = 0.0021, ρ value -0.5556; HIV-1_BaL_ gp120 and HIV-1_SF162_: P = 0.0003, ρ value -0.6322) (Fig 6B and 6C). Noteworthy, when we analyzed at the median signal intensity across the gp140 cluster (clades A, B, C, D, and F) the correlation between binding and neutralization was even more pronounced (P < 0.0001, ρ value -0.6760), indicating enhanced neutralization by cross clade specificity (Fig 6D).

## Discussion

During the last decades, a major focus in HIV-1 research has been directed towards the isolation of broadly neutralizing antibodies [34, 36–39]. Recent improvements in antibody screening platforms and antibody isolation techniques from HIV-1 infected patients have led to the discovery of many potent and broadly neutralizing antibodies [40–42]. Binding specificity assays and standardized neutralization assays [33, 34] are used to understand antibody breadth and activity. Furthermore, growth in this research activity, demands development of assays that maximize the use of sampled material, while reducing the number of samples for multi-parameter measurements. In fact, the National Institute of Allergy and Infectious Diseases provides an AIDS/HIV specimen repository, where particular scrutiny is placed on requests to use limited human specimens with clinical background from infants, paired mother-to-child transmission cases, newly seroconverted individuals, and long-term non-progressors.

In order to streamline HIV-1 vaccine discovery and simultaneously evaluate binding against many different envelope proteins, more efficient and high throughput methods are needed. Here, we developed a novel high throughput technology for characterizing antibodies. We printed selected HIV-1 envelope proteins of different clades onto a protein microarray chip. Using this technique we were able to evaluate five recombinant gp140 envelope proteins, four gp120 envelope proteins, one gp41 protein, and a total of 5 MPER peptides, two of which were in a putative trimer stalk conformation (09128 and C22-pT) [43]. The recombinant gp140 proteins were primarily produced in CHO cells, except HIV-1_SF162_ gp140, which was produced in HEK293 cells. For the expression of the gp120 constructs HEK293 cells and insect cells were used. No information could be found for the expression of HIV-1_96ZM651_ gp120. Therefore, the variety of expression hosts could in some cases explain differences in binding, due to possible differences in glycosylation patterns or other differences in the production of proteins in different cells [44]. For example, the glycan dependent mAb 2G12 exhibited a strong signal (i.e. 40 times higher) against the HIV-1_CN54_ gp120 construct expressed in insect cells, compared to the HIV-1_CN54_ gp140 construct expressed in CHO cells. In contrast, VRC01 or 447-52D revealed only minor differences in signal intensity between the two constructs, probably indicating differences in the glycosylation pattern of the different host systems. However, the gp140 cluster should allow for a robust cross clade comparison, as four out of five constructs were expressed in CHO cells.

Our chip binding analysis revealed extensive cross clade reactivity of V3 loop specific mAbs 447-52D and F425 B4e8 against envelope proteins of the gp140 and the gp120 cluster. Both antibodies are placed among the more broadly neutralizing V3 antibodies and share a similar binding epitope [45]. In the case of mAb 447-52D, it is the well-conserved GPGR motif at the tip of the V3 loop of clade B isolates, thus increasing the neutralization and binding breadth compared to other V3 loop specific antibodies [46]. In contrast, more recently discovered broadly neutralizing variable loop specific mAbs, like the V1/V2 specific mAb PG9, require highly complex glycan dependent quaternary epitopes [47, 48]. A moderate to high degree of cross reactivity against gp140 and gp120 envelopes was also visible with the potent and broadly neutralizing mAb VRC01, within the group of CD4bs antibodies. Interestingly, next to VRC01, the rather poorly neutralizing CD4bs mAb F105 exhibited a high degree of binding specificity against the gp140 cluster. This observation is in good agreement with other studies showing high reactivity of mAb F105 against a broad range of viruses despite having poor neutralization capability [49, 50].

Interestingly, some of the tested gp120 specific mAbs revealed poly-reactivity against gp41, including mAbs F425 B4e8 and F105 (Fig 1). Signal intensities of the latter mAbs measured on the chip followed a dilution curve. However, the signals turned into noise after the mAbs reached a concentration of 1.0 μg/mL. In sharp contrast, mAb 2G12 only exhibited minimal signal at the highest antibody concentration. A similar trend was found with the gp41 specific antibody 240-D, which revealed reactivity towards HIV-1_BaL_ gp120. Interestingly, we did not observe poly-reactivity of F105 and 2G12 against HIV-1_JRFL_ gp41 in our ELISA approach. However, mAb F425 B4e8 showed a slightly elevated OD_450_ at the highest antibody concentration (10 μg/mL) tested. It is important to note that the protein density on the ELISA is about 15 times less than protein density on the microarray chip, thus explaining some of the poly-reactivity observed. MPER specific mAbs 2F5 and 4E10 reacted predominantly with gp41 and MPER analogs and with minor extent to HIV-1_SF162_ gp140.

MPER analog C22-pT was proposed to be in a prefusion state where critical residues for 2F5 and 4E10 binding are buried within the interface of the helices [25]. This was in good agreement with our in-solution-binding assay where 2F5 and 4E10 minimally reacted with the C22-pT wild type peptide. However, when we introduced a N674D mutation into C22-pT (09128), binding was substantially increased particularly for mAb 4E10 (more than 80 fold enhancement in IC_50_ compared to control peptide 94.1). This could be due to enhanced exposure of critical 2F5 and 4E10 contact residues within the trimeric stalk. Although our experimental setup mostly enriches for enhanced 2F5 and 4E10 binding by down selecting those peptides that bind known MPER antibodies, it has to be pointed out that this selection may not be ideal if the method is to be used to capture binding by unknown specificities, where a larger number of antigens may add more relevant information.

To further assess the role of the MPER prefusion state on antibody recognition and subsequent neutralization, a D674N mutation identical to the C22-pT protein was introduced into HIV-1 isolates JRFL and JR-2 to render the viruses more resistant to neutralization. Pseudotyped viruses (wild-type and D674N mutants) were tested against several gp41 and gp120 specific antibodies in a single round infectivity assay. The improved neutralization observed with 2F5 and 4E10 (S2 Table) was a rather unexpected result, in light of the observations of Liu and colleagues [25]. However, our results could be explained by conformational changes in the MPER due to increased flexibility at the hinge region and thus better exposure to MPER specific antibodies in the context of the Env trimer. In contrast, gp41 specific antibody D5 and the gp120 specific antibodies 2G12 and b12 were minimally affected by the point mutation. A negative effect of the D674N mutation was observed with mAb Z13e1, which completely lost neutralization capacity at the highest concentration tested. This is in good agreement with a previous study where D674 has been identified as one of the key residues for Z13e1 binding [21].

Unbiased hierarchical cluster analysis revealed important information about antibody binding characteristics including: 1) antigen specificity, 2) clade specificity, and 3) binding affinity. Moreover, antibodies were grouped into distinct clusters according to their epitope specificity (e.g. gp41 specific antibodies and gp120 specific antibodies), which in our case represents an additional quality control and feature of the microarray chip. This approach applied to unbiased screening of mAbs can be easily adapted in a high throughput, automated manner to efficiently compare panels of well-characterized mAbs against dozens of antigens and rapidly classify uncharacterized mAbs according to antigen binding patterns. In addition, it will rapidly generate actionable data on large numbers of mAbs while consuming small amounts of valuable reagents.

When we first compared our microarray chip data with data obtained through a direct binding ELISA approach, we found a high conformity in binding intensities and OD_450_ values. To compare the two platforms more efficiently we calculated a correlation coefficient between the determined EC_50_ values generated from binding curves. We found a strong correlation between the data of the microarray and the ELISA approach indicating that our chip analysis is a valuable tool for antibody binding characterization.

However, in some cases low correlation exists between the ELISA and chip generated EC_50_ values. For example 447-52D reacted with clade C envelope HIV-1_96ZM651_ gp120 on the ELISA platform but an EC_50_ could not be calculated on the microarray platform. We believe this was most likely due to a combination of antigen accessibility and protein stability over time. We found a higher affinity for HIV-1_96ZM651_ with mAb 447-52D than with VRC01. However, signal intensities of VRC01 against HIV-1_96ZM651_ on the chip were several folds higher than with mAb 447-52D (data not shown). We observed up to 40-fold variation in EC_50_ between the two platforms. Possible factors that could influence the goodness of curve fit and thus EC_50_ calculations were: 1) the antigen concentration spotted onto the chip, 2) the access to antibody specific epitopes, 3) the antibody dilution range, and 4) the overall stability and quality of printed antigens.

Nevertheless, we believe that optimization of this novel technology will yield an excellent tool for comprehensive antibody profiling and characterization in a high throughput manner. For example we envision a high throughput-screening platform for the determination of antibody epitopes by printing a comprehensive set of gp120 mutants or a full set of overlapping peptides covering envelope or distinct regions thereof. To overcome the issues with quaternary structure dependent antibodies such as PG9 or PGT151, and to differentiate between neutralizing and non-neutralizing antibodies, SOSIP trimers [51] could be mutated and printed in a next step. This assay could then be used to determine binding profiles in addition to the characterization of broadly neutralizing antibodies typically done in micro-neutralization assays using very limited quantities of micro-culture wells. Additionally, this technology could be applied to screen clinical samples (plasma and serum) and further our understanding of the evolving humoral immune response among HIV-1 infected persons at different stages of disease and antiretroviral treatment as demonstrated by our results. Last but not least, our chip analysis could be used to evaluate HIV-1 specific antibody profiles among vaccinated individuals from clinical trials, or from animal immunization studies, in a fast and accurate manner. This could be very useful in combination with SOSIP trimers as they allow differentiating between neutralizing and non-neutralizing immune responses.

## Supporting Information

S1 Fig Binding epitopes of all HIV-1 specific mAbs used for microarray probing. Antibodies are sorted according to their neutralization classification and epitope recognition. Epitopes of non-envelope specific control antibodies are also indicated.
(TIF)

S2 Fig In solution competition ELISA with synthesized MPER analogs. Isoleucine zipper motif (IZM) stabilized trimeric MPER peptides C22-pT (N674) and 09128 (D674) were tested for mAb 2F5 (A) and 4E10 binding (B) in an in solution competition assay. Linear MPER peptides 09129, 09122, and 08023 were also examined for 2F5 (C) and 4E10 (D) reactivity. Peptides IZM and 94.1 were used as control peptides. Each curve is representative of at least two independent experiments performed in duplicate.
(TIF)

S3 Fig HIV-1 envelope specific mAbs bind gp140 proteins with high affinity. Binding curves of HIV-1 gp120-specific mAbs F425-B4e8, 2G12, VRC01, and gp41-specific mAb 4E10 were generated from two independent measurements. Different concentrations of antibody (i.e. half log dilutions starting at 10 μg/mL) were titrated on microarray chips with printed HIV-1 envelope antigens. The dissociation constant, Kd for each antibody-antigen interaction was determined using GraphPad PRISM 5.0 one-site binding algorithm and the corresponding affinity constants (1/Kd) calculated.
(TIF)

S1 Table Clade specificity and expression hosts of all recombinant HIV-1 envelope proteins used for chip generation. All proteins except gp41 were obtained through the NIH AIDS Reagent Program. Clade specificity and expression host systems are indicated. HIV-1_SF162_ gp140 was expressed as a trimer.
(DOCX)

S2 Table Neutralization capacity (IC_50_ in μg/mL) of gp41 and gp120 specific antibodies after site directed mutagenesis at MPER amino acid position 674 (D to N mutation). MAb Den 3 was used as a negative control. Neutralization assays were performed at least twice.
(DOCX)
