# Supplementary figures and images for: A High Throughput Protein Microarray Approach to Classify HIV Monoclonal Antibodies and Variant Antigens
Source: PLoS One. 2015 May 4;10(5):e0125581. doi: 10.1371/journal.pone.0125581 (PMC4418728; doi:10.1371/journal.pone.0125581)

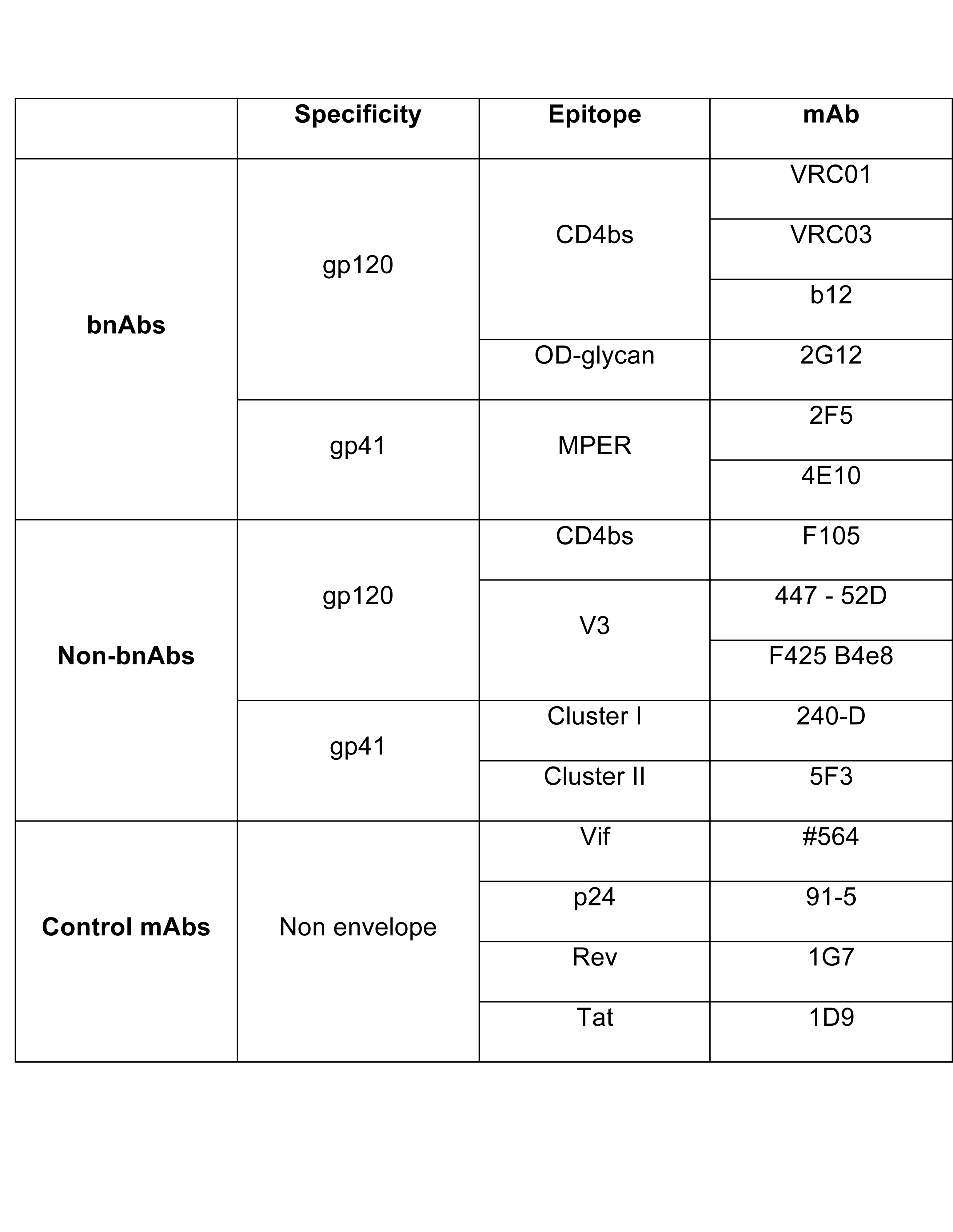

Supplement: S1 Fig — Antibodies are sorted according to their neutralization classification and epitope recognition. Epitopes of non-envelope specific control antibodies are also indicated. (TIF) [file pone.0125581.s001.tif]

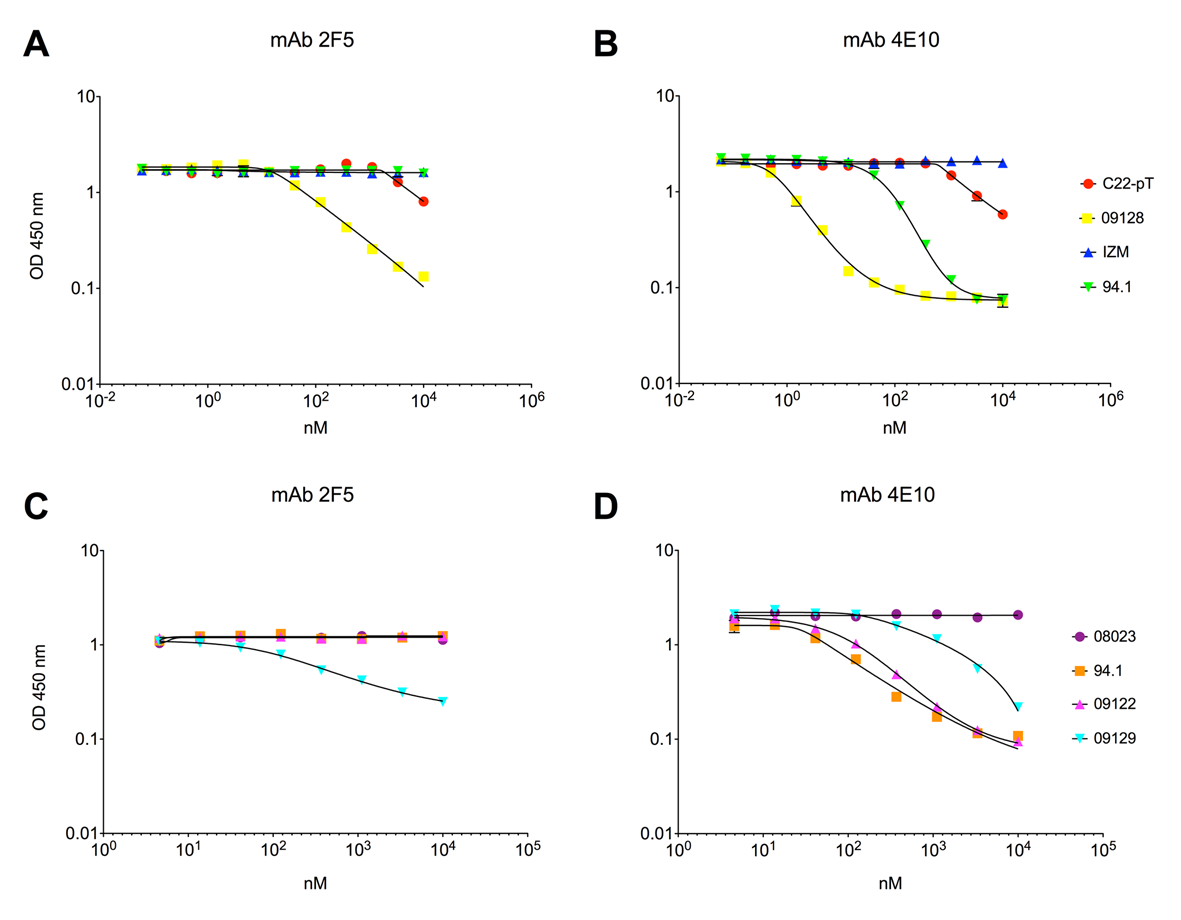

Supplement: S2 Fig — Isoleucine zipper motif (IZM) stabilized trimeric MPER peptides C22-pT (N674) and 09128 (D674) were tested for mAb 2F5 (A) and 4E10 binding (B) in an in solution competition assay. Linear MPER peptides 09129, 09122, and 08023 were also examined for 2F5 (C) and 4E10 (D) reactivity. Peptides IZM and 94.1 were used as control peptides. Each curve is representative of at least two independent experiments performed in duplicate. (TIF) [file pone.0125581.s002.tif]

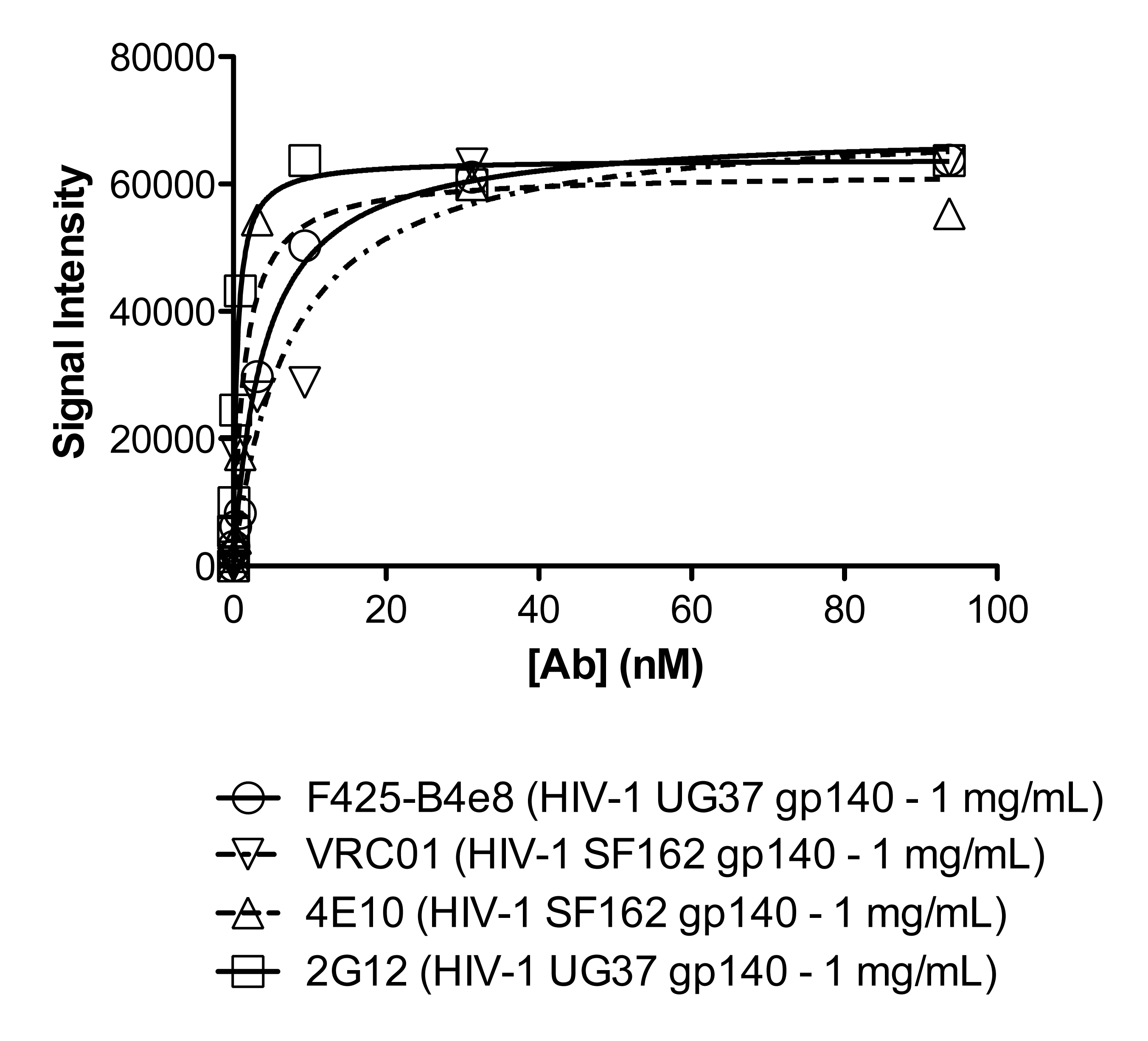

Supplement: S3 Fig — Binding curves of HIV-1 gp120-specific mAbs F425-B4e8, 2G12, VRC01, and gp41-specific mAb 4E10 were generated from two independent measurements. Different concentrations of antibody (i.e. half log dilutions starting at 10 μg/mL) were titrated on microarray chips with printed HIV-1 envelope antigens. The dissociation constant, Kd for each antibody-antigen interaction was determined using GraphPad PRISM 5.0 one-site binding algorithm and the corresponding affinity constants (1/Kd) calculated. (TIF) [file pone.0125581.s003.tif]
