# Supplementary material for: A High Throughput Protein Microarray Approach to Classify HIV Monoclonal Antibodies and Variant Antigens
Source: PLoS One. 2015 May 4;10(5):e0125581. doi: 10.1371/journal.pone.0125581 (PMC4418728; doi:10.1371/journal.pone.0125581)
Supplement: S1 Table — All proteins except gp41 were obtained through the NIH AIDS Reagent Program. Clade specificity and expression host systems are indicated. HIV-1SF162 gp140 was expressed as a trimer. (DOCX) [file pone.0125581.s004.docx]

**Table S1.** HIV-1 envelope proteins printed on the chip. All proteins except gp41 were obtained through the NIH AIDS Reagent Program. Clade specificity and expression host systems are indicated. HIV-1_SF162_ gp140 was expressed as a trimer. All other proteins were expressed as monomers.

| HIV-1 isolate | Env subunit | Clade | Expression host |
| --- | --- | --- | --- |
| UG37 | gp140 | A | CHO |
| SF162 | gp140 | B | HEK293 |
| CN54 | gp140 | C | CHO |
| UG21 | gp140 | D | CHO |
| BR29 | gp140 | F | CHO |
| BaL | gp120 | B | HEK293 |
| CN54 | gp120 | C | Insect cells |
| 96ZM651 | gp120 | C | - |
| 93TH975 | gp120 | E | Insect cells |
| JR-FL | gp41 | B | E. coli |
