# Supplementary material for: A High Throughput Protein Microarray Approach to Classify HIV Monoclonal Antibodies and Variant Antigens
Source: PLoS One. 2015 May 4;10(5):e0125581. doi: 10.1371/journal.pone.0125581 (PMC4418728; doi:10.1371/journal.pone.0125581)
Supplement: S2 Table — MAb Den 3 was used as a negative control. Neutralization assays were performed at least twice. (DOCX) [file pone.0125581.s005.docx]

**Table S2.** Neutralization capacity (IC_50_ in μg/mL) of gp41 and gp120 specific antibodies after site directed mutagenesis at MPER amino acid position 674 (D to N mutation). MAb Den 3 was used as a negative control. Neutralization assays were performed at least twice.

| Antibody | HIV-1_JR-2_ | HIV-1_JR-2 (D674N)_ | HIV-1_JR-FL_ | HIV-1_JR-FL (D674N)_ |
| --- | --- | --- | --- | --- |
| **2F5** | 10.7 | 1.4 | 4.7 | 1.0 |
| **4E10** | 18.1 | 4.9 | 6.4 | 3.1 |
| **Z13e1** | 67.9 | >100 | 35.6 | >100 |
| **2G12** | 0.4 | 0.6 | 0.5 | 0.4 |
| **b12** | <0.4 | <0.4 | <0.4 | <0.4 |
| **D5** | >100 | >100 | >100 | >100 |
| **Den3** | >100 | >100 | >100 | >100 |
